# Cross sectional study on attitudes of Serbian mothers with preschool children: should a HIV-positive female teacher be allowed to continue teaching in school?

**DOI:** 10.1186/s12914-015-0069-4

**Published:** 2015-11-17

**Authors:** Zorica Terzic-Supic, Milena Santric-Milicevic, Momcilo Mirkovic, Svetlana Karic, Ivan Soldatovic

**Affiliations:** Institute of Social Medicine, Faculty of Medicine, University of Belgrade, Belgrade, Serbia; Department for Preventive Medicine, Faculty of Medicine, University of Pristina - Kosovska Mitrovica, Kosovska Mitrovica, Serbia; Preschool Teacher Training College, Sabac, Serbia; Institute of Medical Statistics and Informatics, Faculty of Medicine, University of Belgrade, Belgrade, Serbia

**Keywords:** HIV, Attitude, Stigma, Mother, Women, Preschool children

## Abstract

**Background:**

HIV/AIDS continues to be a serious challenge to public health and human rights in the new millennium. The objective of this survey was to identify the correlation between socio-demographic characteristics and knowledge, attitudes and practices of mothers with preschool children, and their attitude towards whether a HIV-positive female teacher should be allowed to continue teaching in school.

**Method:**

This survey was additional study analysis of the Multiple Indicator Cluster Survey (MICS) in the Republic of Serbia conducted in the period November–December 2010 following the UNICEF methodology. Women deemed eligible for the survey were those who had children under five, had never lost a child, were not pregnant at the time of inquiry and who had a clear attitude (“yes” or “no”) towards whether a HIV-positive female teacher should be allowed to continue teaching in school. The criteria were met by 2309 out of 2992 interviewed women. Pearson chi-square and *t*-test were used to analyse the differences in respondents’ attitude towards whether a HIV-positive female teacher should be allowed to continue teaching in school. Variables that were significantly associated with the dependent variable (*p* < 0.05) were entered into a multiple logistic regression model.

**Results:**

The respondents who were more likely to think that a HIV positive teacher should not be allowed to teach in school were those: who did not know that a healthy-looking person can be HIV-positive (OR = 1.84; 95 % CI = 1.19–2.83), who would not buy (OR = 29.90; 95 % CI = 22.52–39.71) or did not know/were not sure (OR = 2.21; 95 % CI = 1.46–3.33) whether they would buy vegetables from a HIV-positive vendor and women who did not know/were not sure (OR = 2.97; 95 % CI = 1.64–5.39) whether they would take care of a family member sick with AIDS in their own home.

**Conclusion:**

Misconceptions about HIV transmission represent a major barrier to combating HIV/AIDS epidemic and HIV/AIDS-related stigma. It is, therefore, necessary to continue education and raising awareness of human rights both among the population living with HIV and the general population.

## Background

Human immunodeficiency virus infection/acquired immunodeficiency syndrome (HIV/AIDS) is still a serious challenge to public health and society in general, despite notable progress in information, diagnostics, treatment and prevention of the disease in the last two decades [1–3].

Knowledge, Attitude and Practice studies (KAP studies) on HIV/AIDS provide an insight into the current knowledge, attitudes and practices [4] of the general population or of a particular population group, help establish ‘educational diagnosis’ of a community [5] and constitute a basis for planning activities aimed at the prevention and control of HIV [6]. KAP studies also show differences in knowledge and practice, can increase the level of knowledge about HIV/AIDS, and can be a powerful tool to encourage positive attitudes and safe practices in the general population [7, 8]. These studies can also indicate the level of challenge to be faced in the implementation of intervention strategies [8].

Although there is now greater understanding of the disease and its transmission than in past decades, efforts to reduce stigma have shown only moderate success [9]. A number of studies show that stigmatisation – the phenomenon where a person or a group is considered socially unacceptable – is much more pronounced when it is associated with HIV/AIDS than when it is related to other conditions such as mental illness or other physiological health problems [10–12]. Combating stigma and discrimination of persons living with HIV is one of the five imperatives that must be put into practice to ensure sustainability of the initiatives aimed at curbing HIV/AIDS epidemic [13].

Various factors contribute to the stigmatisation of HIV-positive persons, including lack of understanding and knowledge about the disease, misconceptions about HIV transmission, inaccessibility of treatment, the way in which the media report on the epidemic, AIDS incurability, prejudices and fear of certain population groups such as men who have sex with men, injecting drug users, commercial sex workers and other vulnerable groups such as prisoners [14–16]. It is believed that social inequality and the imbalance in economic and political power reinforce stigma [17].

Advances in health care, increasing life expectancy of HIV-positive persons, as well as the fact that HIV/AIDS has become a chronic rather than a fatal disease, have resulted in a desire among persons living with HIV to continue to work [18]. However, along with social stigmatisation, these people face abuse by co-workers and discriminatory behaviour which can result in job-loss leading to unfavourable socio-economic status and inability to meet one’s own basic needs [11, 19, 20].

A limited number of intervention studies targeting HIV/AIDS stigma reduction indicated that people living with HIV face stigma in the workplace [14, 21–23]. Most of the studies of HIV/AIDS related stigma in the workplace have been conducted in developed countries and only a small number of surveys and interventions in underdeveloped or developing countries such as Kenya and Zambia [21–23].

Serbia is a country with a low prevalence of HIV (0.1 % in 2011); HIV epidemic in Serbia is considered to be at a low level and stable/well controlled [24, 25]. Since the first case of HIV infection was registered in Serbia in 1985 [26], numerous activities aimed at combating HIV/AIDS have taken place, including the establishment of NGOs such as the Association against AIDS – JAZAS [27], Center for Affirmation of Positive Living – “Q club” [28], Belgrade Center for Human Rights [29], Coalition against Discrimination [30], Union of Serbian organisations protecting persons living with HIV [31], Charitable Fund of the Serbian Orthodox Church “Covekoljublje” (Eng. philanthropy) [32]. Different projects and activities of NGOs have been implemented to strengthen the capacity for HIV/AIDS prevention among vulnerable groups, providing care and support to persons living with HIV, alleviating HIV/AIDS-related stigma and discrimination, and promoting the importance of testing and early detection of HIV followed by quality counseling. Antiretroviral therapy is available to all who need it and is funded by the Republic Health Insurance Fund. The Strategy on HIV Infection and AIDS [31], which is in line with the relevant international and national strategic documents, was adopted in 2012. One of the specific objectives of the Strategy is “creating discrimination and stigmatisation-free environments for persons living with HIV and other vulnerable and marginalised groups” [31].

There have not been many studies dealing with the intersection of HIV-related stigma and gender. In a meta-analysis of demographic correlations with HIV-related stigma among persons living with HIV in North America, only three out of 24 studies examined gender, which suggests a need to explore further this topic [21]. None of the studies carried out in Serbia has been specifically focused on the stigmatisation of HIV-positive women.

Some studies show that social acceptance is particularly important to women living with HIV, given that they rely on social support more than men in the same position [33, 34]. Hence, understanding by other women is particularly important in terms of acceptance and providing support to HIV-positive women.

The objective of this survey was to identify the correlation between socio-demographic characteristics and knowledge, attitudes and practices of mothers with preschool children, and their attitude towards whether a HIV-positive female teacher should be allowed to continue teaching in school.

## Methods

This survey was based on secondary data analysis of a comprehensive Multiple Indicator Cluster Survey (MICS). MICS was conducted in the Republic of Serbia in the period November–December 2010 by the Statistical Office of the Republic of Serbia, with the financial and technical support of the United Nations Children’s Fund (UNICEF).

### Sample design and the sample

The national sample for the Serbian MICS was designed to provide estimates for a large number of indicators on the situation of children, women and young men at the national level, for urban and rural areas, and for four regions: Belgrade, Vojvodina, Sumadija and Western Serbia, and Southern and Eastern Serbia. The urban and rural domains within 25 areas were identified as the main sampling strata, and the sample was selected in two stages. Within each stratum, a specified number of census enumeration areas were selected systematically with probability proportional to size. After a household listing was carried out within the selected enumeration areas, the listed households were divided into households with and without children under five, and a separate systematic sample of households was selected for each group [35]. At the national level, a total of 6885 households were selected: 3650 households with children under five and 3235 households without children under five. Part of the original MICS database referring to women of reproductive age with children under five was used in this survey. Women deemed eligible for the survey were those who had children under five, had never lost a child, were not pregnant at the time of the inquiry and who had a clear attitude (“yes” or “no”) towards whether a HIV-positive female teacher should be allowed to continue teaching in school. The criteria were met by 2309 out of 2992 interviewed women.

The Serbian MICS protocol was approved on the basis of the Memorandum of Understanding between the UNICEF Serbia and the Statistical Office of the Republic of Serbia [36]. Before the survey, every household received the letter that contained relevant details regarding the background and the objectives. Anonymity, confidentiality and privacy of data were explained and guaranteed for all participants in the survey. It was emphasised that the collected data would serve exclusively for statistical analysis, and their publication would be made only in the aggregate (summary) form by certain demographic and economic categories [37]. Prior face-to-face interview, it was emphasised that all the information “will remain strictly confidential, and answers will never be shared with anyone than our project team” [35]. The informed consent of participants was noted in the questionnaire. The undisclosed personal data were obtained from the UNICEF.

### Study instrument and variables

The data were obtained through face-to-face interviews using the Questionnaire for Individual Women, which was administered to women aged 15–45 years. The questionnaire included 13 modules that contained questions about socio-demographic characteristics of the respondents; child mortality; sexual behavior; and knowledge, attitude and practice about HIV transmission.

The independent variables (yes/no, do not know/not sure/depends answers) included the socio-demographic characteristics (age, region, urban/rural area, marital status, educational level and welfare index), sexual behaviour (use of condom first time they had sex), knowledge (such as, healthy-looking person can be HIV-positive, HIV cannot be transmitted e.g., by mosquito bite, supernatural means), attitude (such as, willingness to take care of a family member sick with AIDS in one’s own home and not to keep the HIV status of a family member a secret), and practice towards persons living with HIV/AIDS (such as, sharing food with a HIV-positive person or buying fresh vegetables from a HIV-positive vendor). According to their attitude towards whether a HIV-positive teacher should be allowed to continue teaching in school or not, the respondents were divided into two groups.

### Statistical analysis

The dependent variable in the study was the question ‘Should a HIV-positive female teacher be allowed to continue teaching in school?’ The independent variables were socio-demographic characteristics, knowledge, attitude and practice towards persons living with HIV/AIDS. Variable data were presented as frequencies (%) for categorical variables and means ± standard deviation (SD) for interval variables. Pearson chi-square and t- test were used to analyse the differences in respondents’ attitude towards dependent variable according to respondents’ characteristics, knowledge and practices, and their attitudes towards HIV/AIDS. All variables that were significantly associated with the dependent variable (*p* < 0.05) were included in a multiple logistic regression model (18 variables out of 19). Multivariate regression modeling was performed in two steps. In the first step, univariate significant socio-demographic variables were included in the model (Enter method). The second step (Forward stepwise method) included practice, knowledge and attitude variables. All p values less than 0.05 were considered significant. All statistical analysis were performed using SPSS 20 (IBM corp.).

## Results

The number of women participating in the survey of the attitudes of Serbian women towards whether a HIV-positive female teacher should be allowed to continue teaching in school was 2309. The average age of the surveyed women was 31.85 ± 6.75.

### Assessment of knowledge, attitudes and practices

All the surveyed women (2309) had heard of AIDS. Out of that number, 1411 (62.4 %) of them used a condom the first time they had sex. The majority of the respondents knew that the risk of HIV transmission can be significantly reduced by having one faithful uninfected partner – 2062 (89.3 %) and by using a condom each time – 2123 (92.0 %). The respondents were generally aware that HIV/AIDS cannot be transmitted by supernatural means (2199 or 95.2 %), by mosquito bite (1726 or 74.8 %) or by sharing food with a HIV-positive person (1735 or 75.4 %). Also, 1907 (82.6 %) knew that a healthy or healthy-looking person can be HIV-positive. When it comes to mother-to-child transmission, the majority of the respondents knew that a mother can transmit the HIV to her baby during pregnancy (2022 or 87.6 %), delivery (1871 or 81.0 %) and through breastfeeding (1652 or 71.5 %). Only 823 (35.6 %) respondents would buy fresh vegetables from a HIV-positive vendor and 896 (38.8 %) would keep the HIV status of a family member a secret, whereas 2145 (92.9 %) would take care of a family member sick with AIDS in their own home. Finally, 1142 (49.5 %) respondents agreed that a HIV-positive female teacher should be allowed to keep teaching in school, whilst the remaining 1167 (50.5 %) that she should not be allowed to do so.

A statistically significant difference between the respondents’ socio-demographic characteristics, age, region and area (urban vs. rural) in which they live, educational level and wealth index quintile, was reflected in their attitude towards whether a HIV-positive female teacher should be allowed to continue teaching in school (Table 1). Respondents who thought that a HIV-positive female teacher should continue teaching in school were older women, 50 % of them lived in Belgrade, AP Vojvodina and urban areas throughout Serbia. Two-thirds of them had post-secondary education and were in households within the richest wealth index quintile. Approximately the same number of respondents living with and without a partner believed that the teacher should continue teaching. Around 80 % of respondents with only primary education and living in households within the poorest wealth index quintile thought that a HIV-positive teacher should not be allowed to continue teaching in school.

**Table 1:** Socio-demographic variables in relation to the respondents’ attitude towards whether a HIV-positive female teacher should be allowed to continue teaching in school

Socio-demographic variables^a^		Should a HIV-positive female teacher be allowed to continue teaching in school?		*p* value
		Yes	No	
Age		32.20 ± 6,16	31.52 ± 7.27	0.016^b^
Region	Central Serbia	553 (44.7)	685 (55.3)	<0.001^c^
	Belgrade	202 (56.9)	153 (43.1)	
	AP Vojvodina	387 (54.1)	329 (45.9)	
Area	Urban	726 (55.3)	588 (44.7)	<0.001^c^
	Rural	416 (41.8)	579 (58.2)	
Currently married or living with a partner	Yes	1066 (49.4)	1094 (50.6)	0,696^c^
	No	76 (51.0)	73 (49.0)	
Level of education	Primary	72 (22.7)	245 (77.3)	<0.001^c^
	Secondary	643 (47.1)	723 (52.9)	
	Higher/faculty	427 (69.2)	190 (30.8)	
Wealth index quintiles	Poorest	106 (31.6)	229 (68.4)	<0.001^c^
	Second Middle	176 (39.1)	274 (60.9)	
	Middle	228 (49.1)	236 (50.9)	
	Fourth	266 (54.8)	219 (45.2)	
	Richest	366 (63.7)	209 (36.3)	

^a^Results are presented as Mean ± SD or n (%), ^b^T test, ^c^Chi-square test

The statistically significant difference between the attitudes and knowledge and practice of the respondents about HIV/AIDS was reflected in their attitude towards whether a HIV-positive teacher should be allowed to teach (Table 2). The negative attitude had respondents who did not use a condom the first time they had sex, and those who did not know that HIV transmission can be avoided by having one faithful uninfected sex partner. and by using condoms. The negative attitude was also expressed by those who did not know that HIV/AIDS cannot be transmitted by supernatural means, by sharing food with a HIV-positive person and by mosquito bite, and by those who did not know that a healthy-looking person can be HIV-positive. More than 80 % of these respondents would not buy fresh vegetables from a HIV-positive vendor and would not allow a HIV-positive teacher to keep teaching in school. Around two-thirds of the respondents would want to keep the HIV status of a family member a secret and would not allow a HIV-positive teacher to continue teaching in school. Half of the respondents who would be willing to take care of a family member sick with AIDS in their own home, they would allow a HIV-positive teacher to continue teaching.

**Table 2:** Practice and knowledge of the respondents in relation to their attitude towards whether a HIV-positive female teacher should be allowed to continue teaching in school

Practice and knowledge about HIV/AIDS		Should a HIV-positive female teacher be allowed to continue teaching in school?		*p* value^b^
		Yes	No	
		N (%)	N (%)	
Used condom the first time they had sex	Yes	497 (57.3)	371 (42.7)	<0.001
	No	630 (44.7)	778 (55.3)	
	DK	13 (65.0)	7 (35.0)	
The risk of HIV transmission can be significantly reduced by having one faithful uninfected partner	Yes	1054 (51.1)	1008 (48.9)	<0.001
	No	69 (48.6)	73 (51.4)	
	DK	19 (18.1)	86 (81.9)	
The risk of HIV transmission can be significantly reduced by using condom each time	Yes	1093 (51.5)	1030 (48.5)	<0.001
	No	32 (42.1)	44 (57.9)	
	DK	16 (14.7)	93 (85.3)	
HIV can be transmitted by supernatural means	Yes	11 (36.7)	19 (63.3)	<0.001
	No	1125 (51.2)	1074 (48.8)	
	DK	6 (7.5)	74 (92.5)	
HIV can be transmitted by mosquito bite	Yes	77 (40.5)	113 (59.5)	<0.001
	No	927 (53.7)	799 (46.3)	
	DK	137 (35.1)	253 (64.9)	
HIV can be transmitted by sharing food with a HIV-positive person	Yes	75 (25.9)	215 (74.1)	<0.001
	No	1008 (58.1)	727 (41.9)	
	DK	55 (19.9)	222 (80.1)	
A healthy-looking person can be HIV positive	Yes	1036 (54.3)	871 (45.7)	<0.001
	No	43 (33.3)	86 (66.7)	
	DK	63 (23.3)	207 (76.7)	
HIV can be transmitted from a mother to her baby during pregnancy	Yes	1024 (50.6)	998 (49.4)	<0.001
	No	34 (55.7)	27 (44.3)	
	DK	84 (37.2)	142 (62.8)	
HIV can be transmitted from a mother to her baby during delivery	Yes	956 (51.1)	915 (48.9)	<0.001
	No	56 (58.9)	39 (41.1)	
	DK	130 (37.9)	213 (62.1)	
HIV can be transmitted from a mother to her baby through breastfeeding	Yes	822 (49.8)	830 (50.2)	<0.001
	No	144 (64.9)	78 (35.1)	
	DK	176 (40.5)	259 (59.5)	
Would you buy fresh vegetables from a HIV-positive vendor?	Yes	740 (89.9)	83 (10.1)	<0.001
	No	234 (18.5)	1030 (81.5)	
	DK^a^	168 (75.7)	54 (24.3)	
Would you want to keep HIV status of a family member a secret?	Yes	428 (41.9)	594 (58.1)	<0.001
	Ne	497 (55.5)	399 (44.5)	
	DK^a^	217 (55.5)	174 (44.5)	
Would you take care of a family member sick with AIDS in your home?	Yes	1107 (51.6)	1038 (48.4)	<0.001
	No	7 (21.2)	26 (78.8)	
	DK^a^	28 (21.4)	103 (78.6)	

*DK*– Do not know, *DK* ^a^ - Do not know/Not sure/Depends, ^b^Chi square test

The predictors of the attitude towards whether a HIV-positive female teacher should be allowed to continue teaching in school were shown in Table 3. The respondents with higher level of education were 0.570 times more likely to think that a HIV-positive female teacher should be allowed to continue teaching in school. The participants who thought that a HIV-positive female teacher should be allowed to continue teaching in school were more likely to know that HIV cannot be transmitted by sharing food with an infected person (0.582 times more),, who would not keep the HIV status of a family member a secret (0.689 times), and who did not know that HIV can be transmitted from a mother to her baby during delivery (0.451 times). The respondents who did not know that a healthy-looking person can be HIV-positive were 1.840 times more likely to think that a HIV-positive teacher should not be allowed to teach. The respondents who would not buy fresh vegetables from a HIV-positive vendor were 30 times more likely to think that a HIV-positive teacher should not be allowed to teach in school. The likelihood for not allowing the teacher to teach was 2.2 times higher for those who did not know/were not sure whether they would buy vegetables from a HIV-positive vendor and was 2.9 times more likely for respondents who did not know/were not sure whether they would take care of a family member sick with AIDS in their own home.

**Table 3:** Multiple logistic regression model: attitude towards should a HIV-positive female teacher not be allowed to continue teaching in school as a dependent variable

Independent variable	P	OR	OR (95 % CI)	
			lower	upper
Education level	<0.001	0.570	0.451	0.719
Age	0.079	0.984	0.966	1.002
Wealth index	0.639	1.024	0.926	1.133
Region				
Central Serbia (reference category)		1^a^		
Belgrade	0.177	1.207	0.918	1.587
AP Vojvodina	0.083	1.411	0.956	2.083
Used condom the first time they had sex				
Yes		1		
No	0.140	1.212	0.939	1.563
DK/do not remember	0.214	0.459	0.134	1.569
HIV can be transmitted by sharing food with a HIV-positive person				
Yes		1		
No	0.004	0.582	0.403	0.843
DK	0.125	1.518	0.890	2.589
A healthy-looking person can be HIV-positive				
Yes		1		
No	0.133	1.526	0.879	2.649
DK	0.005	1.840	1.198	2.827
HIV can be transmitted from a mother to her baby during delivery				
Yes		1		
No	0.008	0.451	0.251	0.809
DK	0.848	1.035	0.727	1.474
Would you buy fresh vegetables from a HIV-positive vendor?				
Yes		1		
No	<0.001	29.902	22.516	39.709
DK/not sure/depends	<0.001	2.205	1.460	3.332
Would you want to keep HIV status of a family member a secret?				
Yes		1		
No	0.005	0.689	0.532	0.894
DK/not sure/depends	0.014	0.649	0.459	0.916
Would you take care of a family member sick with AIDS in your home?				
Yes		1		
No	0.713	1.202	0.450	3.209
DK/not sure/depends	<0.001	2.977	1.641	5.399

1^a^ - reference category

## Discussion

The lack of knowledge and the misconceptions about HIV/AIDS and its transmission make a significant percentage of the population susceptible to the disease. Adequate knowledge and awareness of a disease are the key prerequisites for its prevention and control, given that adequate knowledge is a basis for adopting the appropriate attitudes and practices [38]. It is estimated that 53 % of Serbian women aged 15–49 have comprehensive knowledge about HIV/AIDS [36]. However, women are in a less favourable position compared to men when it comes to education, employment, income and sexual relationships [39]. Many studies show that social acceptance is important to women living with HIV, given that they rely on social support more than men in the same position [33, 34, 40]. Hence, understanding by other women is particularly essential in terms of acceptance and providing support to HIV positive women.

Compared to the knowledge about HIV/AIDS of women at the national level [35], a higher percentage of women included in this survey knew that a healthy-looking person can be HIV-positive and that HIV transmission can be prevented by having one faithful uninfected sex partner as well as by using a condom every time. A similar percentage knew that HIV/AIDS cannot be transmitted by supernatural means, by sharing food with a HIV-positive person and by mosquito bite [35]. Awareness about mother-to-child HIV transmission is important in the context of ensuring that women undergo testing when they conceive and thus contribute to the prevention of HIV transmission to the baby [33]. A higher percentage of women included in this survey, compared to the percentage at the national level, knew that a mother can transmit HIV to her baby during pregnancy (87.6 % vs. 82.4 %), delivery (81.0 % vs. 76.2 %) and through breastfeeding (71.5 % vs. 68.2 %). The most common discriminatory attitude spotted in this survey, as well as at the national level, was the attitude towards keeping the HIV status of a family member a secret and buying food from a HIV-positive vendor [35].

It is well-known that the lack of knowledge and the misconceptions about HIV transmission, as well as the low level of education, are often sources of fear and negative attitudes towards HIV-positive people [34, 41]. In a national survey conducted between 1991 and 1999 in the USA, one-fifth of adult Americans expressed fear of HIV-positive persons, and roughly one-sixth expressed anger or disgust. The results were similar in 2000 [42]. According to our survey, the respondents with higher level of education, who knew that HIV/AIDS cannot be transmitted by sharing food with an infected person and who would not want to keep the HIV status of a family member a secret, had a positive attitude towards a HIV-positive teacher.

Research shows that increasing knowledge about HIV/AIDS can contribute to the alleviation of HIV/AIDS-associated stigma. It has been noted that families of HIV-positive persons often display initial negative attitudes towards HIV which is altered by means of education, making them more accepting of the family the member living with HIV [43–45].

The women participating in our survey who thought that a HIV-positive female teacher should not be allowed to continue teaching in school were those unaware of the fact that a healthy-looking person can be HIV-positive, who were unsure whether they would buy fresh vegetables from a HIV-positive vendor, as well as unsure whether they would take care of a family member sick with AIDS in their own home. A Kaiser Foundation survey in the USA found that 35 % of the respondents would be uncomfortable if their child had a HIV-positive teacher, whereas 34 % claimed they would be very comfortable with that [46]. People who incorrectly believed that certain activities represent a risk for HIV transmission were much more likely to say they would be uncomfortable working with HIV-positive person [46].

Inequalities in social, economic and political power, typical for Serbia in the period of transition [35], form a fertile ground for stigmatisation [16]. HIV-related stigmatisation is more pronounced in societies in which there are other types of stigmatisation [16]. Despite the numerous activities aimed at combating HIV-related stigma and the satisfactory level of knowledge about HIV transmission (as reported by previous surveys conducted in Serbia), there is still a high percentage of population with discriminatory attitude towards HIV positive persons [32, 35]. A umber of studies show that HIV-positive persons often lose employment because of their status, even when they actively contribute to economic well-being of a company [11, 12, 19, 20, 47]. The existence of this phenomenon has been reported in several studies. In a representative sample of 112 HIV-infected patients in the USA, 6 % lost their job within the first six months of Highly Active Antiretroviral Therapy [48]. Among HIV-seroconverters followed in a French group, 18 % have lost their job during the 2.5 years following HIV infection [49]. The situation is similar in China where 63 % respondents believe that it is not safe to work with a HIV positive person [50]. In the UK, majority would feel comfortable working with a colleague who had HIV and believe that people with HIV deserve the same support and respect as those with cancer [51]. Even though there are no official data for Serbia, Bernays and colleagues [52] have reported that HIV-positive persons have a problem with employment. According to them, “if an employer knows that you are HIV-positive, you will not be given a job, you will be kicked out” and “you will not be given opportunity to work – not here, as this is the Balkans” [52]. In spite of high literacy about how to prevent HIV infection, greater tolerance should be demonstrated towards those who get infected. Otherwise, everyone infected might be seen as irresponsible/reckless/uncaring persons. This may need to be studied with other categories of HIV-infected persons in general.

Several studies show connection among the mental disease, stigma and HIV [18, 53, 54]. Major depressive disorder is three times more common in the HIV-positive population compared to the general population [11, 55, 56]. Conservative values and social norms favor stigmatisation of HIV-infected women, based on their gender and the possibility of conception and transmission of the disease to the baby [56], which makes them reluctant to disclose their HIV status and deprives them of much needed support of the family, friends and community [57]. If the stigma-induced distress was to exceed one’s coping ability [21], then the observed stigmatizing attitudes could lead to adverse psychological outcomes. Such a process needs to be further investigated, particularly among women in Serbia.

Gender inequality, particularly pronounced in developing countries [58, 59], is an additional barrier for eradicating HIV/AIDS-associated stigma. In the context of HIV infection, gender is one of the key predictors of emotional distress in HIV-infected women. This should be taken into account when designing interventions aimed at the improvement of functional status, social support and the level of optimism, which is the subject of well-documented studies [41, 60]. This survey highlights the importance of continuing education (including educational campaigns) about HIV/AIDS transmission and prevention with a view to dispelling myths and misconceptions about HIV/AIDS and encouraging positive attitudes towards persons living with HIV. It is necessary to continue the advocacy against gender inequality, having in mind that women living with HIV are twice as vulnerable because of their sex and their HIV status [61]. Although the Strategy on HIV Infection and AIDS of the Republic of Serbia [32] contains reference to the promotion and protection of the human rights of persons living with HIV and other vulnerable and marginalised groups, it is needed to explicitly focus on HIV-positive women and undertake necessary steps, given that these women have serious existential problems and are constantly avoided and neglected [62].

## Limitations

The study limitation is that the knowledge about HIV was assessed on the basis of misconceptions about HIV transmission, which is only one aspect of knowledge about HIV/AIDS. In addition, the existence of HIV-related stigma was assessed on the grounds of personal attitudes of the respondents. However, the responses and the actual practice do not always match [63]. For example, the awareness of that HIV-positive persons should not be discriminated against, might have led interviewed women to give ‘acceptable’ answers, whereas in practice they actively discriminate against persons with HIV (e.g., in health care provision) [64]. Researchers suggest that a stigmatization of an HIV-positive person should be measured on a specialized scale, e.g., Berger HIV Stigma Scale [65, 66]. Also, we could use other variables as dependent such as willingness to take care of a family member sick with AIDS in one’s own home and willingness to keep a secret about the HIV status of a family member.

Despite the above-mentioned limitations, the standardised instrument was used because it enables comparison of the results of the UNICEF surveys conducted in various countries [67].

## Conclusion

Insufficient knowledge about HIV/AIDS, discriminatory attitudes and uncertainty about the safety of interacting with persons living with HIV influence the decision whether a HIV-positive teacher should be allowed to continue teaching in school.

Creating an accepting environment in which persons living with HIV do not feel threatened and are not perceived as a threat, can be achieved by increasing knowledge and dispelling myths about HIV/AIDS among the general population, as well as by raising the awareness of HIV-positive persons about their human rights and ‘equipping’ them with skills for fighting against stigmatisation.
